# Pedicle screw placement accuracy in robot-assisted versus image-guided freehand surgery of thoraco-lumbar spine (ROBARTHRODESE): study protocol for a single-centre randomized controlled trial

**DOI:** 10.1186/s13063-024-07908-1

**Published:** 2024-02-03

**Authors:** Nicolas Aurouer, Patrick Guerin, Arnaud Cogniet, Nicolas Gangnet, Morad Pedram, Pierre-Thierry Piechaud, ReSurg, Jacobus H. Muller, Mo Saffarini, Sonia Ramos-Pascual, Kinga Michalewska, Paolo Mangione

**Affiliations:** 1 ELSAN Group, Hôpital Privé Saint Martin, Pessac, France; 2 Elsan Group, Clinique St Augustin, Cellule Recherche Clinique Nouvelle Aquitaine, Bordeaux, France; 3 ReSurg SA, Nyon, Switzerland

**Keywords:** Pedicle screw placement accuracy, Thoraco-lumbar, Robotic surgery, Freehand surgery, Spinal arthrodesis, Mazor X Stealth

## Abstract

**Background:**

Robotic spinal surgery may result in better pedicle screw placement accuracy, and reduction in radiation exposure and length of stay, compared to freehand surgery. The purpose of this randomized controlled trial (RCT) is to compare screw placement accuracy of robot-assisted surgery with integrated 3D computer-assisted navigation versus freehand surgery with 2D fluoroscopy for arthrodesis of the thoraco-lumbar spine.

**Methods:**

This is a single-centre evaluator-blinded RCT with a 1:1 allocation ratio. Participants (*n* = 300) will be randomized into two groups, robot-assisted (Mazor X Stealth Edition) versus freehand, after stratification based on the planned number of pedicle screws needed for surgery. The primary outcome is the proportion of pedicle screws placed with grade A accuracy (Gertzbein-Robbins classification) on postoperative computed tomography images. The secondary outcomes are intervention time, operation room occupancy time, length of stay, estimated blood loss, surgeon’s radiation exposure, screw fracture/loosening, superior-level facet joint violation, complication rate, reoperation rate on the same level or one level above, functional and clinical outcomes (Oswestry Disability Index, pain, Hospital Anxiety and Depression Scale, sensory and motor status) and cost-utility analysis.

**Discussion:**

This RCT will provide insight into whether robot-assisted surgery with the newest generation spinal robot yields better pedicle screw placement accuracy than freehand surgery. Potential benefits of robot-assisted surgery include lower complication and revision rates, shorter length of stay, lower radiation exposure and reduction of economic cost of the overall care.

**Trial registration:**

ClinicalTrials.gov NCT05553028. Registered on September 23, 2022

## Introduction

### Background and rationale {6a}

There are many indications for thoracolumbar spinal arthrodesis, including deformities (scoliosis, kyphosis), spondylolisthesis, discopathy and spinal instability. Posterior arthrodesis with pedicle screw and rod placement has established itself as the reference technique since the 1980s. The accuracy of this surgical technique was improved by 2D fluoroscopy and then by 3D computer-assisted navigation (CAN), which made it possible to reduce extra-pedicular placement of screws and subsequent complications [1]. Furthermore, in the 2000s, spinal surgical robots were introduced, and these have evolved over the last decades to allow pre- and intraoperative planning (implant selection, optimal trajectory), robotic guidance and real-time 3D navigation [2] thanks to integrating CAN systems within the robotic platform.

Compared to freehand surgery, robotic surgery may result in better pedicle screw placement accuracy [3–5], reduction in intraoperative irradiation [4] and reduction in the length of stay (LOS) [4]. A meta-analysis by Staartjes et al. [6] revealed significantly fewer revisions for screw malposition following thoracolumbar arthrodesis using robotic or CAN guidance compared to freehand surgery, which may imply an association between pedicle screw placement accuracy and revision rates. However, there are few randomized controlled trials (RCT) that have assessed pedicle screw placement accuracy of the newest generation of spinal robots with integrated CAN.

Pedicle screw placement accuracy is an objective outcome measure that can be assessed immediately following surgery and, unlike patient-reported outcomes, does not depend on patient expectations, co-morbidities or follow-up rates. Pedicle screw placement is also one of the most frequently reported outcomes of spinal arthrodesis and facilitates comparisons of surgical methods across the literature.

### Objectives {7}

The objectives are to compare pedicle screw placement accuracy on computed tomography (CT) images of robot-assisted surgery with integrated CAN versus conventional freehand surgery with 2D-fluoroscopy in patients requiring arthrodesis of the thoracic and/or lumbar spine and to compare intervention time, operation room (OR) occupancy time, LOS, estimated blood loss (EBL), surgeon’s radiation exposure, screw fracture/loosening, superior-level facet joint violation, complication rate, reoperation rate on the same level or one level above, functional and clinical outcomes (Oswestry Disability Index [ODI] [7], pain, hospital anxiety and depression scale [HAD], sensory and motor status) and cost-utility analysis.

### Trial design {8}

This is a single-centre evaluator-blinded RCT in a superiority framework with a 1:1 allocation ratio. There will be eight investigating surgeons, each of them capable of performing either intervention (robot-assisted and conventional). All the surgeons have equal experience performing robot-assisted arthrodesis and at least 5 years of experience performing conventional spine arthrodesis.

## Methods: participants, interventions and outcomes

### Study setting {9}

The study is based in the Hôpital Privé Saint Martin, ELSAN Group, Allée des tulipes, 33600 Pessac, France.

### Eligibility criteria {10}

Three hundred patients undergoing posterior arthrodesis of the thoracic and/or lumbar spine will be enrolled.

The inclusion criteria will be as follows:

1. Men and women aged over 18 years,
2. Any indication requiring arthrodesis of the thoracic and/or lumbar spine,
3. Signature of informed consent prior to any study-related procedures,
4. Ability to answer questionnaires and to communicate freely in French,
5. Negative urinary pregnancy test,
6. Affiliation to social security scheme.

The exclusion criteria will be as follows:

1. Psychological disorders,
2. Dependence on analgesics,
3. Chronic infection,
4. History of instrumented thoraco-lumbar surgery,
5. Adult under guardianship, curatorship or other legal protection, deprived of liberty by judicial or administrative decision,
6. Body mass index (BMI) greater than or equal to 40 kg/m^2^
7. Pregnant, postpartum, or breastfeeding women,
8. Participation in another clinical trial.

### Who will take informed consent? {26a}

Participants in the study will sign the written informed consent which will be collected by the investigating surgeon, in accordance with the rules of Good Clinical Practice for biomedical research (Journal Officiel de la République Française n° 0277, decision of November 24, 2006, article 4.8.3).

### Additional consent provisions for collection and use of participant data and biological specimens {26b}

Not applicable.

### Interventions

#### Explanation for the choice of comparators {6b}

Patients will be randomized into two groups, robot-assisted versus conventional, with a 1:1 allocation after initial stratification based on the planned number of pedicle screws needed for surgery, with short-segment fusion comprising four to six screws (two to three vertebrae) and long-segment fusion comprising at least eight screws (more than three vertebrae).

The robot-assisted group will undergo posterior thoracic and/or lumbar arthrodesis using a new-generation surgical robot with integrated real-time 3D navigation (Mazor X Stealth Edition). The conventional group will undergo posterior thoracic and/or lumbar arthrodesis using the freehand surgical technique with 2D fluoroscopy.

#### Intervention description {11a}

Patients will undergo spinal arthrodesis with a pedicle screw placement by posterior approach under general anaesthesia. The robot-assisted group will be operated using a spinal robot with integrated real-time 3D navigation (Mazor X Stealth Edition). All patients in the robot-assisted group will require a CT scan as part of preoperative assessment, to be used for surgical planning and intraoperative navigation. The conventional group will be operated using the freehand surgical technique with 2D fluoroscopy. Surgical access will be open or minimally invasive, depending on the investigating surgeon’s preference and the indication for surgery. Interbody fusion will be performed in selected cases, depending on the indication for surgery.

#### Criteria for discontinuing or modifying allocated interventions {11b}

The investigating surgeon may temporarily or permanently interrupt a patient’s participation in the study for any reason that would serve the best interests of the patient, in particular, in the case of a serious adverse event (SAE). These patients will no longer be monitored under this protocol but will continue to receive the best care possible given their state of health and the current state of knowledge. For all early study withdrawals, the investigating surgeon must document the reasons with as much detail as possible. The study may be interrupted prematurely in the event of the occurrence of unexpected adverse events (UAE). Similarly, unforeseen events or new information regarding medical strategies may lead the sponsor (Hôpital Privé Saint Martin ELSAN) to terminate the study prematurely. Modifications should be documented by the investigating surgeon. Only dropouts result in the discontinuation of follow-up. Even in the event of a deviation from the protocol, the follow-up of the participant must be carried out until the end specified in the protocol.

#### Strategies to improve adherence to interventions {11c}

To improve adherence to interventions, preoperative CTs will be easily accessible for patients allocated to the robot-assisted group, and the necessary surgical equipment will be available at all times for both groups.

#### Relevant concomitant care permitted or prohibited during the trial {11d}

There is no specific concomitant care administered nor prohibited during the trial.

#### Provisions for post-trial care {30}

There is no anticipated harm or compensation for trial participation. Participation in the study will stop 24 months after arthrodesis; patients will undergo routine follow-up.

### Outcomes {12}

The primary outcome of this study will be the rate of pedicle screws placed with grade A accuracy according to Gertzbein-Robbins classification (Table 1) in the robot-assisted versus conventional groups. The evaluation of the placement of the screws will be based on CT images from postoperative day 1 and will be carried out by two radiologists independent to the surgical team, blinded to the technique used for screw placement. In case of disagreement between the two evaluators, a 3rd evaluator will be requested.

**Table 1:** Gertzbein-Robbins classification of pedicle screw placement

Grade A	fully intrapedicular position without breach of the pedicle cortex
Grade B	<2 mm cortical breach
Grade C	2-4 mm cortical breach
Grade D	4-6 mm cortical breach
Grade E	exceeding the pedicle cortex <6 mm or is outside of the pedicle

The secondary outcomes of the study will be as follows:

1. Intervention time (min)
2. OR occupancy time (min)
3. LOS (days)
4. EBL (volume of the suction jar at the end of the intervention in ml)
5. Surgeon’s intraoperative radiation exposure (a dosimeter placed in contact with the surgeon’s clothing, in cGy/cm^2^)
6. Signs of screw fracture/loosening (CT at 12 and 24 months postoperatively)
7. Superior-level facet joint violation according to Babu-Mehta classification (Table 2; CT on postoperative day 1)
8. Types of complications (neurologic, infectious, vascular, general, late and early mechanical complications) and complication rate at 24 months postoperatively
9. Reoperation rate on the same instrumented level or one level above at 24 months postoperatively
10. Functional and clinical outcomes at 3, 12 and 24 months postoperatively (pain on visual analogue scale [VAS; 0, best; 10, worst], ODI [0, best; 100, worst], HAD scale [0, best; 21, worst], sensory status with 3-point scale [0, absent sensation; 2, normal sensation], motor status with Medical Research Council’s [MRC] scale for muscle strength [0, no contraction; 5 full muscle strength])
11. Cost-utility analysis based on medical costs and quality of life (QoL) evaluation (quality-adjusted life years [QALYs] derived from EuroQol [EQ-5D-5L questionnaire) at 12 months postoperatively

**Table 2:** Babu-Mehta classification of facet joint violation

Grade 0	Screw not in facet
Grade 1	Screw in lateral facet but not in facet articulation
Grade 2	Penetration of facet articulation
Grade 3	Screw travels within facet articulation

### Participant timeline {13}

The inclusion will last 24 months. The patient participation, from the surgery day to the end of follow-up, will be 24 months (Table 3).

**Table 3:** Participant timeline

	**Pre-inclusion visit (V0)** **D0 -9 to 120 days**	**Inclusion visit (V1)** **D0 -2 to 90 days**	**Surgery (V2)** **D0**	**3 months follow-up (V3)** **D0 + 90 days ± 30**	**12 months follow-up (V4)** **D0 + 360 days ± 30**	**24 months follow-up (V5)** **D0 + 720 days ± 30**
ENROLMENT:						
Patient information	X					
Eligibility screening		X				
Urinary pregnancy test		X				
Informed consent		X				
Allocation		X				
INTERVENTION:						
Radiation exposure measurement			X			
ASSESSMENT:						
CT scan			X^a^		X	X
Full-spine x-ray or EOS		X		X	X	X
ODI score		X		X	X	X
Pain on VAS		X		X	X	X
HAD scale		X		X	X	X
Sensory status		X		X	X	X
Motor status (MRC)		X		X	X	X
EQ5D-5L questionnaire		X		X	X	
Complications			X	X	X	X
Concomitant treatment		X	X	X	X	X
Adverse events			X	X	X	X

^a^Postoperative day 1

### Sample size {14}

Patients are considered as “clusters” of screws, due to the inter-dependence on the placement of screws in the context of complex cases (e.g. morbid obesity, bone fragility). The required sample size of screws is calculated to compare the proportions of grade A placed screws (Gertzbein-Robbins classification) by robot-assisted versus conventional surgery in thoracolumbar arthrodesis. According to the literature, the proportion of grade A placed screws of conventional surgery is approximately 88% [3, 8], and we hypothesize that robot-assisted surgery will improve this proportion by 7%. Therefore, 725 screws per group are needed to detect a statistically significant difference in the proportion of grade A placed screws, assuming an intracluster correlation coefficient (ICC) of 0.3, with a power of 90% and a bilateral *α* of 5%. Based on the assumption that each patient is implanted with an average of 5 screws [4, 9, 10] and that 5% of patients will be randomized but not operated, the total number of patients to be randomized is 300 (150 patients per group). The sample size was determined using the Power Analysis & Sample Size (PASS) software 2021 (NCSS).

### Recruitment {15}

Recruitment will be carried out within the Department of Orthopedic Spine Surgery of Hôpital Privé Saint Martin in Bordeaux/Pessac. Patients with indications for arthrodesis of the thoracic and/or lumbar spine will be offered participation in the study during the V0 consultation where the information note will be delivered by the investigating surgeon.

About 500 patients per year are seen for this surgery within the establishment, and we estimate that at least 150 of them are likely to meet the inclusion criteria. The recruitment is estimated at 24 months.

### Assignment of interventions: allocation

#### Sequence generation {16a}

Before randomization, patients will be stratified according to the planned number of screws needed for surgery, with short-segment fusion comprising four to six screws (two to three vertebrae) and long-segment fusion comprising at least eight screws (more than three vertebrae). The randomization sequence will be generated before the commencement of the study separately for each stratum with the R software and will be integrated into the data management software (REDCap).

#### Concealment mechanism {16b}

The randomization sequences for each stratum will be concealed from the staff as well as from the patients.

#### Implementation {16c}

The patient will be randomized to a treatment arm only after inclusion, and the allocation will be based on a random number attributed to the patient’s electronic case report form (e-CRF), which is only accessible by the study staff.

### Assignment of interventions: blinding

#### Who will be blinded {17a}

This is an evaluator-blinded study, in which both the investigating surgeon and participant know which treatment is being administered. The only people blinded to the treatment arm are the two radiologists evaluating pedicle screw placement on postoperative CT images.

#### Procedure for unblinding if needed {17b}

The design is open-label with only outcome evaluators being blinded, so unblinding will not occur in any circumstances.

### Data collection and management

#### Plans for assessment and collection of outcomes {18a}

The following datasets will be defined:

1. Intention-to-treat (ITT; effect of assigning the treatment): This dataset will include all enrolled patients who undergo surgery, who will be analysed in their assigned groups, irrespectively of possible protocol deviations or crossovers between treatment arms.
2. Per-protocol (PP; effect of receiving the treatment): This dataset will include enrolled patients who undergo their assigned treatment without major deviations from the protocol (deviations that could impact the assessment of the primary endpoint).

The following information will be included on the e-CRF after enrolment and allocation:

1. Patients’ characteristics: age (years), sex, weight (kg), height (cm) and pregnancy test
2. Radiological data: screw placement accuracy, screw fracture/loosening and superior-level facet joint violation on CT images on postoperative day 1 and at 12- and 24-months follow-up
3. Clinical data: intervention time, LOS, EBL, surgeon’s intraoperative radiation exposure, sensory and motor function at inclusion and all follow-up visits
4. Functional outcomes: pain, ODI, HAD, EQ5D-5L at inclusion and all follow-up visits
5. Medical costs at 12 months follow-up

The primary outcome will be assessed for both datasets (ITT and PP). The secondary outcomes will be assessed on the ITT dataset.

#### Plans to promote participant retention and complete follow-up {18b}

The data for the primary outcome (pedicle screw placement accuracy) will be collected on postoperative day 1, so the risk of missing data is small. For the analysis on the ITT dataset, if the evaluation of the placement of a screw is missing, it will be considered that the screw is not correctly placed. If the evaluation of all screws is missing for one patient, the patient will not be analysed, although the patient will still be included in the initial cohort. All the other data are considered secondary outcomes; the missing data will be reported as such in the final analysis. Outcomes will be collected for all the patients regardless of possible protocol deviations or crossovers between treatment arms.

If a patient withdraws prematurely from the study, they will not be replaced, and reasons for patient withdrawal must be documented. For patients lost to follow-up, attempts to contact the patient must be made and documented. No further outcomes will be collected from the patients who discontinue participation in the study.

#### Data management {19}

The data collected will be taken from each patient file completed by the investigating surgeon during medical visits. They will be then transferred to a secure database (REDCap). Patient data will be anonymized before being entered into the database, assigning each patient a unique ID number, so that duplicates can be checked later. An input mask will be established to initially configure the type of variables to be entered and its modalities. Health data will be collected through a validated e-CRF of REDCap software, meeting Title 21 Code of Federal Regulation Part 11 and hosted on REDCap’s health data certified servers, meeting all the levels of security required by the Food and Drug Administration (FDA), European Medicines Agency (EMA) and in compliance with the General Data Protection and Regulation (GDPR).

#### Confidentiality {27}

In accordance with the legal provisions in force (articles L.1121-3 and R.5121-13 of the Public Health Code [PHC]), persons having direct access to the source data will take all the necessary precautions to ensure the confidentiality of the information relating to experimental drugs, to research, to the patients who take part in it and in particular with regard to their identity as well as to the results obtained. These persons, like the investigating surgeons themselves, are subject to professional secrecy.

During the biomedical research or at its end, the data collected (on the patients who agree to it) and transmitted to the sponsor by the investigating surgeons (or any other specialized stakeholders) will be made anonymous. Under no circumstances should they clearly show the names of the patients concerned or their addresses.

Each patient will be assigned a confidential identification code, made up of the investigating surgeon centre number (two digits), a two-letter code (first name initial + last name initial) and the patient’s inclusion order number in the centre (three digits).

The sponsor will ensure that each patient who agrees to the research has given their written consent for access to the individual data concerning them, which is strictly necessary for the quality control of the research.

#### Plans for collection, laboratory evaluation and storage of biological specimens for genetic or molecular analysis in this trial/future use {33}

Not applicable as no biological samples will be collected for future analysis.

## Statistical methods

### Statistical methods for primary and secondary outcomes {20a}

The quantitative variables will be described by the number of values entered, the number of missing data, the mean, the standard deviation, the median, the 1st and 3rd quartiles, the minimum and the maximum. The qualitative variables will be described by the number of values entered, the number of missing data, the frequency, the percentage of each modality and the 95% confidence interval (CI) of each modality, if relevant. The type I error (*α*) is set at 5% with a two-tailed analysis for the entire study. No management of multiplicity will be carried out. The secondary outcomes will be analysed on an exploratory basis. The primary outcome (proportion of grade A placed screws according to Gertzbein-Robbins classification on postoperative day 1) will be described by group (robot-assisted vs. conventional surgery) and compared using a marginal logistic model (taking into account screw clusters in the same patient) including the group and the randomization stratification factor (long-segment fusion vs. short-segment fusion). The results of the marginal logistic model will be interpreted in terms of adjusted odds ratio (aOR) accompanied by the 95% CI. Intervention time, OR occupancy time, LOS, EBL and surgeon’s intraoperative radiation exposure will be described by group (robot-assisted vs. conventional surgery) and compared using an analysis of variance (ANOVA) including the group and the randomization stratification factor (long-segment fusion vs. short-segment fusion). Radiological stability (signs of screw fracture/loosening), superior-level facet joint violation, complication and reoperation rates will be compared using logistic regression including the group and the stratification factor of the randomization (long-segment fusion vs. short-segment fusion). Functional outcomes (ODI, pain, HAD) will be compared using an analysis of covariance (ANCOVA) including the group, the score at inclusion and the randomization stratification factor (long-segment fusion vs. short-segment fusion); this analysis will be carried out at each evaluation time (3-, 12- and 24-month follow-up). The evaluation of sensory status and motor status will be compared using a Cochran-Mantel-Haenszel test including the group and the stratification factor of the randomization (long-segment fusion vs. short-segment fusion); this analysis will be carried out at each evaluation time (3-, 12- and 24-month follow-up). The cost-utility analysis will be carried out according to the recommendations of the French National Authority for Health (HAS) [11], based on QALYs (lifespan weighted by health-related quality of life), derived from the EQ5D-5L. All direct medical costs will be measured over 12 months following hospital discharge. The incremental cost-utility ratio (difference in costs divided by the difference in QALYs) between robot-assisted surgery and conventional surgery will be determined at 12 months along with their 95% CIs. All statistical analysis will be performed using the latest version of the SAS software.

### Interim analyses {21b}

No interim analyses are planned. Given that both conventional and robot-assisted interventions are already validated in clinical practice and in the literature, there is no reason to anticipate exceptionally poor outcomes or unexpected complications. Furthermore, reaching a statistically significant difference between the groups before the planned end of the study is unlikely with the anticipated cohort size. Additionally, interim analyses could lead to false conclusions, as shown by Armitage et al. [12].

### Methods for additional analyses (e.g. subgroup analyses) {20b}

The analysis of the subgroups of this study will be based on the stratification factor (long-segment fusion vs. short-segment fusion).

### Methods in analysis to handle protocol non-adherence and any statistical methods to handle missing data {20c}

There is no method for non-adherence to the protocol, as the risk for non-adherence is negligible.

### Plans to give access to the full protocol, participant-level data and statistical code {31c}

Apart from the publication of the English version of the protocol, the full protocol, dataset and statistical code will not be made publicly available.

The following documents relating to this research are archived in accordance with the Good Clinical Practice (GCP) guidelines and the regulations in force by the investigating surgeons and/or the sponsor for a period of 15 years following the end of the research:

- Participant source folders (only the surgeons),
- The protocol and any amendments to the protocol,
- All other documents and letters relating to the research.

Furthermore, the medical file is kept according to the usual regulatory archiving provisions.

No displacement or destruction can be carried out without the agreement of the sponsor. At the end of the regulatory archiving period, the sponsor will be consulted for destruction of the files. All data, documents and reports are subject to audit or inspection.

## Oversight and monitoring

### Composition of the coordinating centre and trial steering committee {5d}

The research will be regulated by the standard operating procedures of Cellule Recherche Clinique Nouvelle Aquitaine ELSAN under the supervision of the main author (Dr. Nicolas Aurouer). The Cellule Nouvelle Aquitaine is created by the study sponsor and is responsible for providing centralized monitoring of the study, in accordance with Good Clinical Practices (I.C.H. version 4 of May 1, 1996, and decision of November 24, 2006). The Cellule is composed of a scientific director (medical coordination, scientific advisor), a clinical study coordinator (planning, management, monitoring and study coordination) and a clinical research associate (patient monitoring and data entering). There is a weekly exchange of information between the clinical research associate and the clinical study coordinator. In case of any doubt or disagreement, the scientific director is consulted.

### Composition of the data monitoring committee, its role and reporting structure {21a}

There will be no data monitoring committee, as this is a category 2 study (article L1121-1 of the PHC: interventional research on human subjects, involving only minimal risks and constraints), which corresponds to research evaluating usual care, hence, not anticipating new adverse events, overwhelming benefit, nor futility.

### Adverse event reporting and harms {22}

Adverse event (AE) is defined in article R.1123-39 of the PHC as any harmful manifestation occurring in a person who undergoes biomedical research, regardless if this manifestation is linked to the research or to the product to which this research relates.

SAE is defined in the same legal article as any AE that:

- Leads to death,
- Endangers the life of the person who agrees to the research,
- Requires hospitalization or extension of hospitalization,
- Causes a significant or lasting incapacity or handicap,
- Results in a congenital anomaly or malformation,
- Is considered medically serious.

UAE is defined in the same article and concerns the research involving health products. It is any undesirable effect of the product whose nature, severity or evolution does not match the information given in the user manual.

The investigating surgeon must assess each AE and record it in the patient’s medical file and in the observation notebook.

In the context of a category 2 study (PHC article L1121.1: interventional research on human subjects, involving only minimal risks and constraints), the implementation of specific vigilance (such as vigilance unit, safety committee) is not necessary. The occurrence of an unexpected SAE or a new fact will follow the usual vigilance process with the Regional Pharmacovigilance Centres (CRPV) or the National Agency for the Safety of Medicines and Health Products (ANSM) depending on the nature of the SAE.

There are no expected SAEs related to the study itself. However, SAEs related to the surgeries are expected:

1. During the procedure or associated with the surgery:
  - Surgical site infection
  - Breach of the dura mater, pseudo-meningocele, persistent cerebrospinal fluid leak, meningitis and subdural hematoma
  - Root or spinal cord neurological deficit: sensory and/or motor (dysesthesia, hyperesthesia, anaesthesia, hyperpathia, paresis or paralysis of one or more muscle groups) and genito-sphincter disorders (urinary and/or faecal incontinence, cauda equina syndrome, impotence, perineal and/or sexual insensitivity, sexual dysfunction)
  - Compressive epidural haematoma
  - Parietal or retro-peritoneal haematoma
  - Visual deficit due to ocular compression related to the installation on the operating table
  - Mechanical complications: spontaneous vertebral fractures with or without neurological disorders, deformation in kyphosis, mobilization of the material and pseudarthrosis (lack of consolidation of the arthrodesis)
  - Junctional syndrome (degradation of a level adjacent to the arthrodesis): degenerative stenosis, herniated disc, deformation in kyphosis, posterior joint degeneration, rapidly destructive disc disease, spondylolisthesis and laterolisthesis
  - Digestive disorders: ileus, gastritis and intestinal obstruction
  - Urinary infection and/or acute retention of urine related to urinary catheterization
  - Respiratory infection
  - Thromboembolic complications: phlebitis and pulmonary embolism
  - Lesion of the large pre-spinal vessels
  - Change in mental status
  - Death
2. Associated with the device:
  - Loosening of implants
  - Disassembly of the device
  - Screw or rod fracture
  - Allergy to implant components
  - Metallosis
  - Formation of a tumour related to implant debris or triggering of an autoimmune disease
  - Painful conflict of the implants with the skin
  - Bad positioning of the implants inducing neurological, vascular, intervertebral disc or posterior articular lesions
  - Loss of correction of spinal deformity

The occurrence of a pregnancy during the research period does not constitute a SAE. However, any pregnancies must be notified to the vigilance unit according to the same procedures as a SAE because it will require a specific follow-up. Any anomaly noted on the foetus or the child will be recorded. Any voluntary termination of pregnancy, therapeutic termination of pregnancy or miscarriage must be notified and transmitted according to the same procedures as a SAE.

### Frequency and plans for auditing trial conduct {23}

A clinical research associate mandated by the sponsor will visit the investigating centre on a regular basis, during the setting up of the research, one or more times during the research depending on the rhythm of inclusions and at the end of the research.

During these visits, the following elements will be reviewed:

- The presence of a copy of the informed consent, completed and signed
- Compliance with the research protocol and the procedures defined therein
- The quality of the data collected in the observation notebook: completeness, accuracy, missing data and consistency of the data with the source documents (medical records, appointment books, original laboratory results, etc.)
- Maintenance of on-site documentation
- The reporting of unexpected SAEs or new facts to the vigilance centre

A report will be written after each visit.

An audit may be carried out at any time by persons appointed by the sponsor, independent of the research managers. Its objective is to ensure the quality of research, the validity of its results and compliance with the law and regulations in force. The investigating surgeons agree to comply with the sponsor’s requirements for an audit and the competent authority for research inspection. The audit may apply to all stages of the research, from the development of the protocol to the publication of the results and the classification of the data used or produced in the context of the research.

### Plans for communicating important protocol amendments to relevant parties (e.g. trial participants, ethical committees) {25}

Any substantial modification (i.e. any modification likely to have a significant impact on the protection of persons, on the conditions of validity, on the results of the research and on the interpretation of the scientific documents which support the progress of the research or the methods of conducting it) will be the subject of a written amendment which will be submitted to the sponsor; prior to its implementation, the latter will obtain a favourable opinion from the Comité de Protection des Personnes (CPP). If necessary, non-substantial modifications (i.e. those having no significant impact on any aspect of the research whatsoever) will be communicated to the CPP for information.

All amendments to the protocol must be brought to the attention of all investigating surgeons participating in the research, who will respect their content.

Any amendment that modifies the care of participants or the benefits, risks and constraints of the research is subject to a new information note and a new consent form, the collection of which follows the same procedure as the one mentioned above.

### Dissemination plans {31a}

Pursuant to Law No. 2002-303 of March 4, 2002, relating to the rights of patients and the quality of the health system, amended by Law No. 2016-41 of January 26, 2016, relating to the modernization of the health system, participants have a right of access, during and at the end of the study, to their medical file. The research file constitutes a communicable element of the patient’s medical file, according to the terms of article L 1111-7 of the PHC. In accordance with Law No. 2002-303 of March 4, 2002, participants are informed, at their request, of the overall results of the research.

The final research report will be written by the coordinator and the biostatistician. This report will be submitted to each of the investigating surgeons for their opinion. Once a consensus has been reached, the final version must be endorsed by the signature of each of the investigating surgeons and sent to the sponsor as soon as possible after the actual end of the research. A report must be transmitted to the competent authority as well as to the CPP within one year, after the end of the research, understood as the last follow-up visit of the last subject included. This period is reduced to 90 days in the event of premature termination of the research.

All the data collected during this research are the property of the sponsor of the study and cannot be communicated in any case to a third person without the written agreement of the sponsor. The results will be submitted to peer-reviewed journals and presented at national and international conferences.

## Discussion

According to most recent meta-analyses, freehand surgery with 2D fluoroscopy provides worse pedicle screw placement accuracy compared to robot-assisted surgery and computer-assisted navigation (CAN) [13–18], with robot-assisted surgery granting the best results [14, 19]. However, some meta-analyses have found comparable outcomes for freehand versus robot-assisted surgery [20–22].

During the last decade, four spinal robots with integrated CAN (ROSA ONE Spine, ExcelsiusGPS, Mazor X Stealth Edition, TiRobot) have been introduced into the market, allowing for surgical planning, guidance, and real-time 3D navigation [2]. Of these four robots, only TiRobot (TINAVI) has been evaluated in RCTs [3, 23–25], providing excellent outcomes, although this robot is uniquely available on the Chinese market. Furthermore, two recent meta-analyses have suggested the advantage of integrating CAN into a robotic system [21, 26]. Therefore, it is important to further investigate the combination of these two technologies. Additionally, there is a scarcity of evidence concerning the economic aspect of using spinal robots, both during the intervention and in the first 12 months following hospital discharge. The latter could include painkiller consumption, work incapacity, readmissions and revisions. The present RCT will provide high-quality clinical data concerning the Mazor X Stealth Edition and will deliver a cost-utility analysis.

## Trial status

Protocol version number and date: 2022-A00874-39, version 1.0 of 02/06/2022.

Recruitment start date: 26/12/2022.

Estimated recruitment end date: 26/12/2024.

## Data Availability

The sponsor is responsible for obtaining the agreement of all the parties involved in the research in order to guarantee direct access to all the places where the research is carried out, to the source data, to the source documents and to the reports for quality control and audit. The investigating surgeons will make available to the people in charge of monitoring the study, the documents and individual data strictly necessary for follow-up, quality control and audit.

## Abbreviations

AE: Adverse effect
ANSM: Agence Nationale de Sécurité du Médicament et des produits de santé (National Agency for the Safety of Medicines and Health Products)
BMI: Body mass index
CAN: Computer-assisted navigation
CNIL: Commission Nationale de l'Informatique et des Libertés (National Comission for Information Technology and Civil Liberties)
CPP: Comité de Protection des Personnes (research ethics committee)
CRA: Clinical research associate
CRPV: Centre Régional de PharmacoVigilance (regional pharmacovigilance centre)
CT: Computed tomography
EBL: Estimated blood loss
e-CRF: Electronic case report form
EMA: European Medicines Agency
FDA: Food and Drug Administration
GDPR: General Data Protection and Regulation
HAD: Hospital Anxiety and Depression score
HAS: Haute Autorité de Santé (French National Authority for Health)
ICC: Intracluster correlation coefficient
ICH GCP: International Conference on Harmonisation Good Clinical Practice
ITT: Intention-to-treat
LOS: Length of stay
MRC: Medical Research Council
PHC: Public Health Code (Code de la Santé Publique)
PP: Per-protocol
RCT: Randomized controlled trial
ODI: Oswestry Disability Index
OR: Operating room
SAE: Serious adverse event
QoL: Quality of life
QALY: Quality-adjusted life year
UAE: Unexpected adverse event
VAS: Visual analogue scale
